# Classical and intermediate monocytes and their ratios in the natural immune course of ischemic stroke

**DOI:** 10.3389/fnins.2026.1889912

**Published:** 2026-09-30

**Authors:** Marta Oses, María Araceli García-Torres, Cristina Serrano del Castillo, Ana Moraga, Inmaculada Navas, Marta Guillán, Ivana Zamarbide, Víctor Mora, Carolina Peña-Martínez, Fernando Ostos, Patricia Calleja, María Ángeles Moro, Alicia García-Culebras, Ignacio Lizasoain

**Affiliations:** 1 PhD Program in Neuroscience, Universidad Autónoma de Madrid-Cajal Institute, Madrid, Spain 28029; 2 Stroke Unit, Department of Neurology, Hospital Universitario Fundación Jiménez Díaz, Madrid, Spain; 3 Health Research Institute-Fundación Jiménez Díaz University Hospital, Universidad Autónoma de Madrid (IIS-FJD, UAM), Madrid, Spain; 4 Department of Immunology, Hospital Universitario Fundación Jiménez Díaz, Madrid, Spain; 5 Department of Cell Biology and Histology, Faculty of Medicine, Complutense University, Madrid, Spain; 6 Instituto de Investigación Hospital 12 Octubre (imas12), Madrid, Spain; 7 Department of Psychology, School of Health Sciences, Rey Juan Carlos University, Madrid, Spain; 8 Neurovascular Pathophysiology, Centro Nacional de Investigaciones Cardiovasculares (CNIC), Madrid, Spain; 9 Stroke Unit, Department of Neurology, Hospital Universitario 12 de Octubre, Madrid, Spain; 10 Neurovascular Research Unit, Department of Pharmacology and Toxicology, Faculty of Medicine, Complutense University, Madrid, Spain

**Keywords:** classical monocytes, intermediate monocytes, ischemic stroke, lymphocyte-to-classical monocyte ratio, lymphocyte-to-intermediate monocyte ratio, lymphocyte-to-monocyte ratio, stroke-associated infection, monocyte subtypes

## Abstract

**Introduction:**

Monocytes contribute to the immune response to ischemic stroke (IS), participating in both acute inflammation and post-ischemic repair. However, classical (cMo), intermediate (intMo), and non-classical (ncMo) monocytes differ in function, and composite indices such as the lymphocyte-to-monocyte ratio (LMR) do not distinguish between them. We characterized the dynamics of monocyte subpopulations and of two subset-derived ratios, the lymphocyte-to-classical monocyte ratio (LMRclas) and the lymphocyte-to-intermediate monocyte ratio (LMRint), in patients with acute IS who did not undergo reperfusion therapy, allowing assessment of the natural immune response.

**Methods:**

We prospectively studied 31 patients and 16 controls, obtaining complete blood counts and/or flow cytometry in patients at admission, 24 h, and 48 h, and relating these findings to the National Institutes of Health Stroke Scale (NIHSS), the modified Rankin Scale (mRS) through 90 days, and the development of stroke-associated infection (SAI). Correlations were assessed using Spearman’s rank correlation coefficient, with Benjamini-Hochberg correction applied within four prespecified families.

**Results:**

Compared with controls, patients showed reduced lymphocyte counts at admission, 24 h, and 48 h, whereas total monocyte counts did not differ at any time point. Within the monocyte compartment, intMo counts increased at 24 h and 48 h, and LMRint decreased at 48 h, while cMo and ncMo counts remained unchanged. A lower LMR at 48 h was associated with greater stroke severity and with poorer functional outcome; higher cMo counts at 48 h and a lower LMRclas at 24 h were associated with poorer outcome at 90 days; and a lower LMRclas at admission was associated with the development of SAI. None of these associations retained significance after correction for multiple comparisons, and neither ratio remained independently associated with 90-day outcome after adjustment for age, sex, and baseline NIHSS.

**Discussion:**

Taken together, these exploratory findings suggest a reorganization of the monocyte compartment after ischemic stroke despite an unchanged total monocyte count, a shift that composite indices cannot capture but that LMRclas and LMRint might reflect. The estimates reported here may serve as a basis for evaluating these ratios in larger, adequately powered cohorts.

## Graphical Abstract

**Figure:**
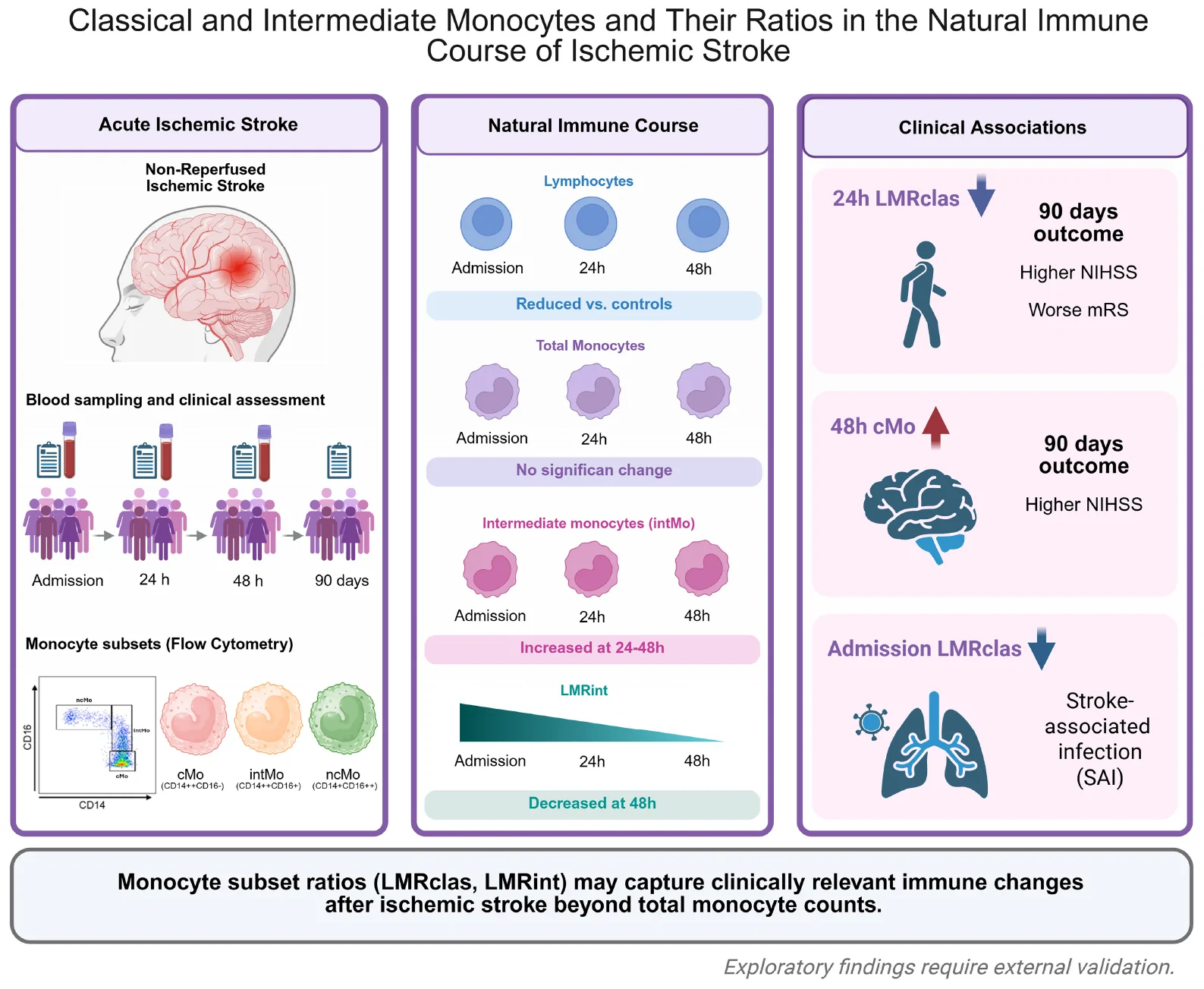
This prospective study characterized monocyte subpopulations in patients with non-reperfused ischemic stroke, sampled at admission, 24 h, and 48 h. Classical monocytes (cMo) at 48 h were associated with greater stroke severity at 90 days. The lymphocyte-to-classical monocyte ratio (LMRclas) at 24 h was associated with poorer functional outcome at 90 days. Lower LMRclas at admission was associated with a higher likelihood of stroke-associated infection during hospitalization. Created with BioRender.com.

## Introduction

Ischemic stroke (IS) is the second leading cause of death and the primary cause of disability in adults in Europe (Soto et al., 2022). Although reperfusion therapies have advanced considerably,

they reach only a minority of IS patients even in well-resourced health systems (Vivanco-Hidalgo et al., 2018; Biswas et al., 2025; Hirsch et al., 2025; Christensen et al., 2026), and fewer than 50% of stroke patients achieve functional independence (Zhao et al., 2024). In patients with minor ischemic stroke (MIS; NIHSS < 5), the benefit of mechanical thrombectomy over best medical treatment has not been established: available observational data show similar 90-day functional outcomes between both strategies (Volny et al., 2020; Safouris et al., 2023; Marto et al., 2025), with thrombectomy associated with a higher rate of symptomatic intracranial hemorrhage (Safouris et al., 2023). This population carries a meaningful disability burden, with up to 30% resulting in poor functional outcome at 90 days (De Santis et al., 2026).

Moreover, an elevated proportion of real-world IS patients (particularly those who are older, lack large-vessel occlusion, or arrive outside the therapeutic window) are managed without reperfusion (Behrndtz et al., 2022).

Early diagnosis and prompt treatment are critical to reducing disability and mortality. However, the presence of subtle or atypical symptoms can result in misdiagnosis and delays in clinical decision-making (Liang et al., 2025). There is an urgent need to identify candidate blood-based indicators that could support clinical assessment, improve patient stratification, and guide decision-making.

Monocytes participate in the pathophysiological response to IS, contributing to the acute inflammatory response and subsequent tissue repair mechanisms (Iadecola et al., 2020).

Human monocytes are conventionally classified into three subtypes based on the differential expression of surface markers CD14 and CD16: classical monocytes (cMo, CD14++CD16-), intermediate monocytes (intMo, CD14++CD16+), and non-classical monocytes (ncMo, CD14+CD16++) (Ziegler-Heitbrock et al., 2010). This classification, the most widely used in clinical practice, reflects not only phenotypic differences but also a sequential ontogenic maturation continuum.

Under physiological conditions, murine studies have shown a stable equilibrium between the proportions of cMo and intMo, with cMo serving as the primary progenitors of intMo (Yona et al., 2013). Upon release from the bone marrow, (including bones such as the skull, as described in mice (Kolabas et al., 2023)) cMo enter the peripheral circulation with a lifespan of approximately 1–2 days in humans (Patel et al., 2017; Cormican and Griffin, 2020). When not recruited to tissues, they may repopulate resident macrophage pools, migrate to reservoir organs, or progressively differentiate first into intMo and subsequently into ncMo, a stepwise process governed by chemokine receptor signaling, most notably through CCR2 (Weber et al., 2000; Patel et al., 2017; Ożańska et al., 2020).

Turning to the pathophysiology of cerebral ischemia, murine models show that the ischemic insult triggers a rapid mobilization of monocytes from peripheral immune reservoirs (such as the spleen and lungs), along with the induction of emergency myelopoiesis (Bao et al., 2010; Kim et al., 2014; Luo et al., 2026). In these models, monocytes are among the first immune cells to infiltrate the central nervous system after IS, typically peaking between 1 and 5 days post-injury before gradually declining over the following week (Ritzel et al., 2015; Wattananit et al., 2016). This initial period is critical, as it coincides with the peak of systemic inflammation and the development of infections, and encompasses one of the highest-mortality periods after IS in humans (Silver et al., 1984).

This functional heterogeneity of monocyte subsets has also been increasingly recognized across multiple disease contexts, with each population displaying distinct and sometimes opposing prognostic significance.

In cardiovascular disease, prospective human flow cytometry (FC) studies have consistently demonstrated independent and divergent prognostic roles for each subpopulation. IntMo independently predicted cardiovascular events at 3-year follow-up in 951 patients undergoing elective coronary angiography (Rogacev et al., 2012). In a cohort of 229 patients with stable coronary artery disease, intMo were associated with major adverse cardiovascular events at 4 years (HR 1.09; 95% CI 1.02–1.16; *p* = 0.006), while cMo were inversely associated with events (HR 0.96; 95% CI 0.94–0.99; *p* = 0.02); the association for intMo persisted after adjustment for age, systolic blood pressure, and left ventricular ejection fraction (Dregoesc et al., 2024). In ST-elevation myocardial infarction, intMo correlated with troponin levels and ventricular function recovery, while cMo correlated with larger infarct scar mass and inversely with ventricular function (Tapp et al., 2012; Bosch et al., 2020). In heart failure, absolute intMo counts independently predicted all-cause mortality alongside reduced cMo levels (Elchinova et al., 2018). In peripheral artery disease, intMo expanded selectively while cMo and ncMo remained unchanged (Wildgruber et al., 2016).

This subset-specific behavior is not confined to vascular disease. In sepsis, intMo expanded, and cMo declined compared with controls (13.6% vs. 6.2% and 72.5% vs. 82.6%, respectively; both *p* < 0.001), with intMo further elevated in culture-confirmed sepsis versus localized infection (17.4% vs. 11.5%; *p* < 0.05) (Greco et al., 2019). In rheumatoid arthritis, the two subsets showed opposite associations with the same clinical scale: intMo correlated positively and cMo negatively with DAS28-ESR disease activity (Tsukamoto et al., 2018). In head and neck squamous cell carcinoma, intMo were selectively reduced and showed increased HLA-G and PD-L1 expression, indicating changes in both function and abundance (Sakakura et al., 2021).

In IS, the few available human studies have likewise reported subset-specific rather than global monocyte responses. In a mixed cohort of ischemic (76%) and hemorrhagic (24%) stroke patients, Urra et al. found that the proportion of cMo remained unchanged after stroke, while intMo increased and ncMo decreased. In models adjusted for age and stroke severity, each 1% increase in cMo was associated with early clinical worsening (OR 1.29), poor 90-day outcome (OR 1.38), and mortality (OR 1.40). In contrast, intMo were inversely associated with mortality (OR 0.32) and ncMo were inversely associated with poor outcome (OR 0.74) (Urra et al., 2009b). The same group subsequently showed that monocyte responses are relevant to the risk of SAI (Urra et al., 2009a), and Kaito et al., in a cohort of exclusively IS patients but using controls with other neurological diseases rather than healthy individuals, reported that intMo were elevated in patients with progressing infarction while ncMo were reduced in those developing SAI (Kaito et al., 2013).

Despite these observations, most clinical studies in IS have relied on total monocyte counts or on immune ratios derived from them, such as the lymphocyte-to-monocyte ratio (LMR), defined as the absolute lymphocyte count divided by the absolute total monocyte count (Dong et al., 2020; Wang et al., 2024). Although a higher total monocyte count has been associated with greater stroke severity and worse prognosis, the phenotypic and functional heterogeneity of monocyte subpopulations highlights the limitations of this approach and the need to clarify the specific contribution of each subset (Greco et al., 2021).

Since cMo, intMo, and ncMo perform distinct pathophysiological functions in the neuroinflammatory and neuroreparative processes that follow IS, total monocyte counts are likely to mask functionally relevant immune dynamics. A subset-based approach could therefore provide a more accurate reflection of the immunological state following cerebral ischemia.

The LMR has been widely used as a prognostic biomarker across multiple conditions, including acute myocardial infarction (Xiong et al., 2025), heart failure (Vakhshoori et al., 2023b), and coronary heart disease (Vakhshoori et al., 2023a). In IS specifically, its prognostic value has been established in a recent systematic review and meta-analysis of six studies including 1,225 patients, in which a lower LMR was significantly associated with poor functional outcome at 3 months, defined as mRS ≥ 3 (OR 0.63; 95% CI 0.49–0.80; *p* = 0.0002) (Tian et al., 2025). Its discriminative capacity, however, remains modest: a reported AUC of 0.672 for 3-month functional outcome is lower than that of the neutrophil-to-lymphocyte ratio in the same cohort (0.703) (Wang et al., 2024).

As a composite index, the LMR treats the monocyte compartment as functionally homogeneous and therefore cannot capture the distinct biological signals contributed by each subset (Ruder et al., 2023). This limitation may partly explain its moderate discriminative performance.

The LMR has established prognostic utility in cerebrovascular disease, and monocyte subset dynamics carry independent biological information that the conventional LMR does not capture. Nevertheless, no study to date has examined whether subset-specific ratios, the lymphocyte-to-classical monocyte ratio (LMRclas, the lymphocyte count divided by the absolute cMo count) and the lymphocyte-to-intermediate monocyte ratio (LMRint, the lymphocyte count divided by the absolute intMo count), provide additional diagnostic or prognostic value in IS. Expressing each subset relative to the lymphocyte count, rather than in absolute terms, integrates the two arms of the post-ischemic immune response, myeloid activation and stroke-induced lymphopenia, into a single index derivable from routine differential counts. This represents a clinically relevant gap, as the identification of patients at risk of poor functional outcome or stroke-associated infection remains an important unmet need in acute stroke care, where early risk stratification could inform more individualized therapeutic and monitoring strategies. Although monocytes constitute a therapeutic immunological target in IS (Elkins et al., 2017; Hernández-Jiménez et al., 2023; Zhang et al., 2026), few human studies have explored the dynamics of each monocyte subtype in IS or their peripheral blood dynamics before transmigration into the central nervous system. In this study, we aimed to explore peripheral blood monocyte subsets and their derived ratios in the natural course of IS.

## Materials and methods

### Study design

This is a prospective, observational clinical study with repeated measurements conducted in accordance with Good Clinical Practice and the Declaration of Helsinki, with written informed consent obtained from participants. The study received approval from the Clinical Research Ethics Committees at Hospital Universitario Fundación Jiménez Díaz and Hospital Universitario 12 de Octubre, both in Madrid, Spain. The project was presented within the framework of the collaborative research network Proyecto Ictus of the GEECV-SEN and RICORS network. A total of 31 patients and 16 healthy volunteers were recruited from the stroke units at Hospital Universitario Fundación Jiménez Díaz and Hospital Universitario 12 de Octubre, between March 2019 and January 2024 (with interruptions in 2020 and 2021 due to the COVID-19 pandemic), according to the inclusion and exclusion criteria detailed below.

Due to the scarcity of prior data on monocyte subset dynamics in IS, formal a priori sample size calculations were not feasible, and the final sample size was determined pragmatically based on the number of eligible patients available during recruitment.

Inclusion criteria: Adult patients aged 18 years or older, previously independent (pre-morbid mRS ≤ 2); with an IS within 24 h of symptom onset who did not receive reperfusion treatment with fibrinolysis or thrombectomy to avoid immunological biases from these procedures (reperfusion facilitates monocyte infiltration into the infarct territory), to study the natural evolution of the processes of cerebral ischemia (Han et al., 2020). In all patients, reperfusion therapy was either not indicated or contraindicated, owing to the absence of large-vessel occlusion, a low NIHSS score with non-disabling symptoms, or presentation outside the therapeutic window.

Twenty-one patients (67.7%) had an NIHSS score of less than 5 at admission, classifying them as MIS according to the conventional definition (Yakhkind et al., 2016).

Exclusion criteria: Patients with IS who received reperfusion treatment, patients with transient ischemic attack, hemorrhagic stroke, subarachnoid hemorrhage, or stroke mimics. Immunosuppressed patients or those with active autoimmune, hematological, or neoplastic disease, as well as those who had any infectious process or hospital admission in the previous month. Patients with a baseline functional dependence (>2 on mRS), pregnant and postpartum women.

Control group: A control group of 16 healthy volunteers (median age, 52.5 years; 31% women) was included. Participants were individuals attending the hospital for routine check-ups who voluntarily donated blood. Complete blood counts were analyzed in 12 participants, and monocyte subpopulations were assessed by FC in 5 participants. Individuals with active infectious, autoimmune, or neoplastic diseases were excluded.

### Clinical data collection

For each patient, we recorded demographic data (age, sex), vascular risk factors (hypertension, diabetes, dyslipidemia, smoking, alcohol consumption, obesity), previous diseases, clinical symptoms, time of onset, duration, topographic location of the lesion, and etiology. Clinical stroke severity was assessed using the NIHSS at admission, 24 h, 48 h, and 90 days.

Functional status was assessed using the modified Rankin scale (mRS) at the same four timepoints (admission, 24 h, 48 h, and 90 days), and the development of stroke-associated infection (SAI) was recorded during hospitalization.

The characteristics of the patients and controls are summarized in Table 1.

**TABLE 1:** Demographics, vascular risk factors and clinical evolution of the study population.

Characteristic	Patients data (*n* = 31)	Controls (*n* = 16)
Age (mean)	73.9 ± 13.1	51.5 ± 8.2
Age (median)	77 (66–84)	52.5 (43–60)
Sex (F:M)	6 (19.4%):25 (80.6%)	5 (31.2%):11 (68.8%)
Vascular risk factors		
Hypertension	21 (67.7%)	—
Diabetes mellitus	12 (38.7%)	—
Dyslipidemia	13 (41.9%)	—
Smoking	10 (32.3%)	—
Alcohol	8 (25.8%)	—
Obesity	5 (16.1%)	—
OSA	4 (12.9%)	—
Heart disease	14 (45.2%)	—
Lung disease	4 (12.9%)	—
Peripheral arteriopathy	3 (9.7%)	—
Prior stroke	8 (25.8%)	—
Dementia	1 (3.2%)	—
Epilepsy	1 (3.2%)	—
Etiology		
Atherothrombotic	12 (38.7%)	—
Cardioembolic	10 (32.3%)	—
Lacunar	3 (9.7%)	—
Undetermined	6 (19.4%)	—
Clinical outcomes		
Recurrence	1 (3.2%)	—
Infection	4 (12.9%)	—
Death at 90 days	2 (6.5%)	—
Scales	Median (IQR)	—
NIHSS at admission	3 (1–5)	—
NIHSS at 24 h	1 (0–5)	—
NIHSS at 48 h	1 (0–5)	—
NIHSS at 90 days	0 (0–1)	—
	**n (%)**	
mRS ≤ 2 at admission	26 (83.8%)	—
mRS ≤ 2 at 24 h	22 (71.0%)	—
mRS ≤ 2 at 48 h	23 (74.2%)	—
mRS ≤ 2 at 90 days	22 (71.0%)	—

The analyzed population consisted of a group of patients with IS (*n* = 31) and a control group (*n* = 16). Twenty-one patients had an NIHSS score of less than 5 at admission, classifying them as MIS (67.7%). Continuous variables are shown as mean ± SD or as median (interquartile range); categorical variables as n (%). IS, ischemic stroke; MIS, minor ischemic stroke; mRS, modified Rankin Scale; NIHSS, National Institutes of Health Stroke Scale; OSA, obstructive sleep apnea; SD, standard deviation.

All patients underwent a baseline acute brain imaging test (CT without contrast and/or multimodal CT) and control imaging tests during hospital admission (CT or MRI). All patients underwent standard complementary tests to identify the potential etiology of the IS. SAI was defined by the presence of the following criteria: body temperature > 37.8°C along with the presence of suggestive clinical symptoms (productive cough, dyspnea, pleuritic pain, urinary tract symptoms), blood leukocyte count > 11,000 cells/μL or < 4,000 cells/μL, pulmonary infiltration in chest X-ray findings, or cultures positive for a pathogen, which became overt within the first 7 days of IS onset (Chamorro et al., 2005).

### Blood samples

Peripheral blood samples were obtained from patients at separate times for complete blood count and FC analysis, to characterize the acute and subacute phases of IS. Complete blood counts were obtained at admission (*n* = 31), 24 h (*n* = 28), and 48 h (*n* = 15). FC samples were obtained at admission (*n* = 31), 24 h (*n* = 8), and 48 h (*n* = 7). An additional complete blood count was obtained at the 90-day follow-up visit (*n* = 20); these variables lie outside the acute-phase focus of the present analyses and are therefore not tabulated individually in Supplementary Tables 2, 3, but were retained in the exploratory family used for the multiple-comparison correction.

During hospitalization, blood samples were drawn between 07:00 and 08:00 to minimize circadian variation.

At each time point, the blood cell count was assessed with an automated hemocytometer. The LMR was calculated as the ratio of absolute lymphocyte to monocyte counts.

For FC studies, total blood samples were stained for cell surface markers using direct immunofluorescence staining-lysis and wash technique, using previously described FC protocols (Van Dongen et al., 2012). The following monoclonal antibody (mAb) combinations were used: mAb anti-CD16-FITC (clone 3G8), mAb anti-CD13-PE (clone L138), mAb anti-CD45-PerCP (clone 2D1), and mAb anti-CD14-APC (clone MφP9) from BD Biosciences (San Jose, CA, United States). Acquisition was performed on a FACSCanto II flow cytometer using the FACSDiva version 8.1 software. Instrument setup and calibration were performed in accordance with the EuroFlow SOP.^1^ A forward scatter (FSC) and side scatter (SSC) dot plot was used to exclude aspirated air and doublets. Analysis was performed using the Infinicyt™ software (Cytognos) and FlowJo LLC software. The identification of monocyte subsets was based on their FSC and SSC characteristics, as well as their antigenic profile. The monocyte subsets were analyzed following the gating strategy illustrated in Figure 1.

**FIGURE 1:**
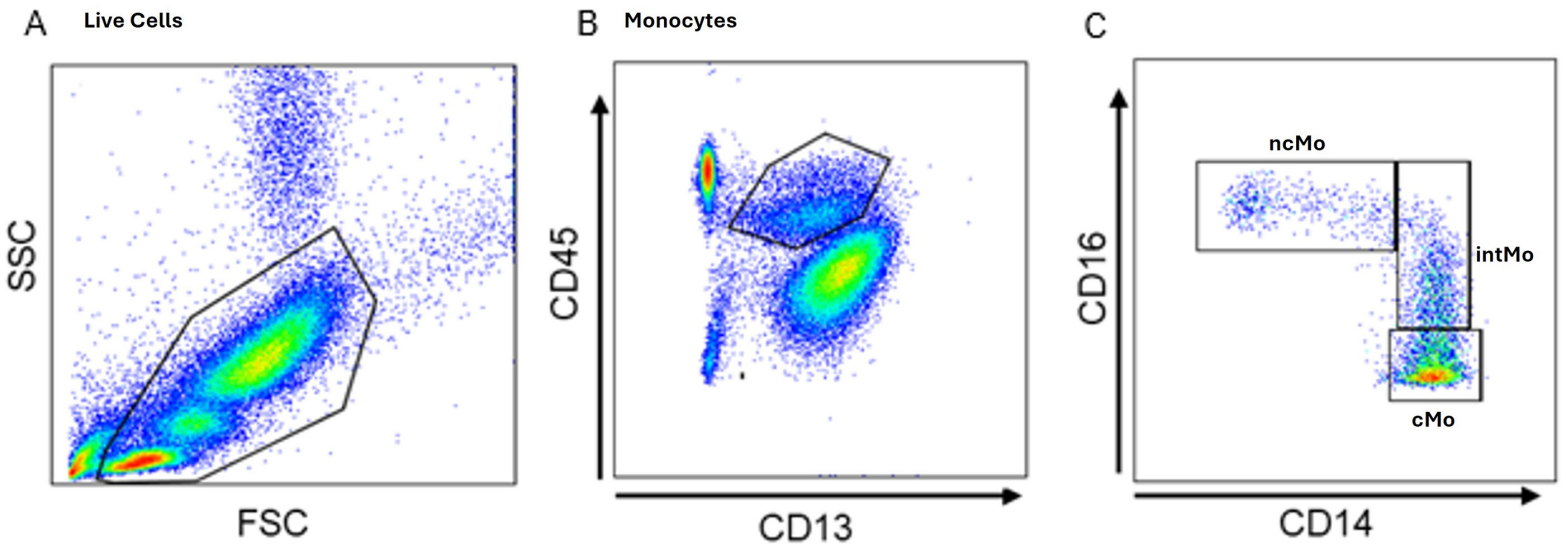
Gating strategy in flow cytometry analysis of blood. **(A)** First, live events are selected in a side scatter (SS) vs. forward scatter (FS) dot plot. **(B)** Monocytes were identified based on their high expression of CD13 and CD45. **(C)** Distinguishing cMo (CD14++CD16-), intMo (CD14++CD16+), and ncMo (CD14+CD16++) monocyte subsets by their respective CD14 and CD16 expression.

Monocytes were identified based on their high expression of CD13 and CD45, and their respective CD14 and CD16 expression distinguished subsets (cMo: CD14++CD16-; intMo: CD14++CD16+; ncMo: CD14+CD16++).

Absolute subset counts were estimated by multiplying the percentage of each subset obtained by flow cytometry by the absolute monocyte count from the automated complete blood count at the same timepoint. Lymphocyte-to-classical monocyte ratio (LMRclas) and lymphocyte-to-intermediate monocyte ratio (LMRint) were calculated as the ratio of lymphocyte to cMo or intMo counts, respectively.

### Data analysis

Quantitative variables were summarized as median and interquartile range (IQR), and qualitative variables as frequencies and percentages. The normality of continuous variables was assessed using the Shapiro-Wilk test. Between-group comparisons were performed using the Mann-Whitney U test for continuous variables and the chi-square test for categorical variables. Comparisons across the three study timepoints (admission, 24 h, and 48 h) were performed using the Kruskal-Wallis test. As only a single control sample was available, this same control group served as the reference for the comparisons performed at each patient timepoint.

Correlation analyses were performed using Spearman’s rank correlation coefficient, given the non-normal distribution of the hematological variables, consistent with the non-parametric approach used throughout the analysis. The mRS was analyzed as a continuous ordinal scale (0–6) throughout the correlation and regression analyses; a dichotomized version (mRS ≥ 3, indicating poor functional outcome) was used only for the prognostic ROC analyses described below. Linear fit lines and their 95% confidence bands are shown for visualization purposes only and do not represent the statistical model used for inference; associations were assessed using Spearman’s rank correlation coefficient.

To quantify the statistical constraints imposed by the available sample, a sensitivity analysis was performed estimating the minimum effect size detectable with 80% power at a two-sided α = 0.05 for each study timepoint, separately for correlational analyses (expressed as minimum detectable | r|, derived from the Pearson coefficient as is conventional for power estimation and interpretable as an approximate lower bound for the rank-based tests used here; see Supplementary Table 1), and for between-group comparisons (expressed as Cohen’s *d*).

Retrospective (*post-hoc*) power calculations were not performed, as this approach is mathematically circular with the obtained *p*-values and does not provide additional inferential information (Hoenig and Heisey, 2001).

*P*-values were adjusted for multiple testing using the Benjamini-Hochberg false discovery rate (FDR) procedure, applied independently within four prespecified analysis families: two primary families for the biomarkers of interest (LMRclas and LMRint), and two exploratory families for the remaining variables. The mRS-primary family comprised the six biomarker variables (LMRclas and LMRint at admission, 24 h, and 48 h), each tested against four mRS assessments (baseline, 24 h, 48 h, and 90 days), yielding 24 comparisons. The NIHSS-primary family comprised the same six biomarker variables, each tested against four NIHSS assessments, yielding an additional 24 comparisons. The mRS-exploratory and NIHSS-exploratory families each comprised the remaining 62 hematological and flow-cytometric variables and derived ratios, tested against the four mRS assessments and the four NIHSS assessments, respectively (62 × 4 = 248 comparisons per family). Both unadjusted and FDR-adjusted (q) values are reported throughout.

Given the limited number of adverse clinical events, adjusted associations between LMRclas, LMRint, and clinical outcome at 90 days were assessed using four parsimonious models: two ordinal logistic regressions on the full modified Rankin Scale (mRS, 0–6) and two linear regressions on 90-day NIHSS as a continuous measure of neurological severity, one of each for LMRclas and LMRint at admission. Results from the ordinal models are expressed as adjusted odds ratios (OR) with 95% confidence intervals; results from the linear models as β coefficients with 95% confidence intervals. In all four models, confidence intervals and *p*-values were obtained by the Wald method. All four models were adjusted for age, sex (coded as male versus female), and baseline NIHSS; additional covariates were not included given the risk of model instability with the available sample size.

Receiver operating characteristic (ROC) curve analyses were performed for all three biomarkers (total LMR, LMRclas, and LMRint) for two purposes: diagnostic discrimination between IS patients and controls, and prognostic discrimination of poor functional outcome (mRS ≥ 3) at 90 days. The area under the curve (AUC) was reported with 95% confidence intervals calculated using the DeLong method, and the optimal cut-off was selected using the Youden index, together with the corresponding sensitivity and specificity.

The association between admission LMRclas and the occurrence of SAI during hospitalization was assessed using logistic regression, with results expressed as odds ratios (OR) and 95% confidence intervals.

Statistical analyses were performed using STATA statistical software (Version 16; StataCorp LP, College Station, TX, United States) and R (R Core Team, 2013; R Foundation for Statistical Computing, Vienna, Austria). Statistical significance was set at a two-sided α of 0.05.

## Results

### Clinical data

A total of 31 patients were enrolled in the study. The median age was 77 years (66–84 years); 19% were women and 81% were men. The main vascular risk factors are described in Table 1, with hypertension being the most frequent. Regarding stroke etiology, 38.7% of strokes were of atherothrombotic origin, 32.3% were cardioembolic, 9.7% were lacunar, and 19.4% were due to undetermined causes. Four patients developed infections during hospitalization, and 1 patient experienced stroke recurrence during the follow-up period. The control group had a median of 52.5 years and included 5 women and 11 men. At 3 months, 71.0% of patients achieved functional independence (mRS ≤ 2; 22 of 31), 9 patients (29.0%) had a poor functional outcome (mRS ≥ 3), and 2 patients (6.5%) died during this period (for more details see Table 1).

### Acute changes in lymphocyte but not total monocyte counts during the natural course of non-reperfused ischemic stroke

Compared with the control group (2250 cells/μL; IQR 1550–2650), patients with IS exhibited significantly lower lymphocyte counts at admission (1500 cells/μL; IQR 1200–1900; *p* = 0.026), at 24 h (1500 cells/μL; IQR 1300–2000; *p* = 0.012), and at 48 h (1400 cells/μL; IQR 1200–1900; *p* = 0.035) (Figure 2A). No significant differences were observed in total monocyte counts at admission (500 cells/μL; IQR 400–700; *p* = 0.31), 24 h (550 cells/μL; IQR 400–700; *p* = 0.14), or 48 h (500 cells/μL; IQR 400–600; *p* = 0.46), compared with controls (400 cells/μL; IQR 400–500), and did not change significantly over time (*p* = 0.732) (Figure 2B).

**FIGURE 2:**
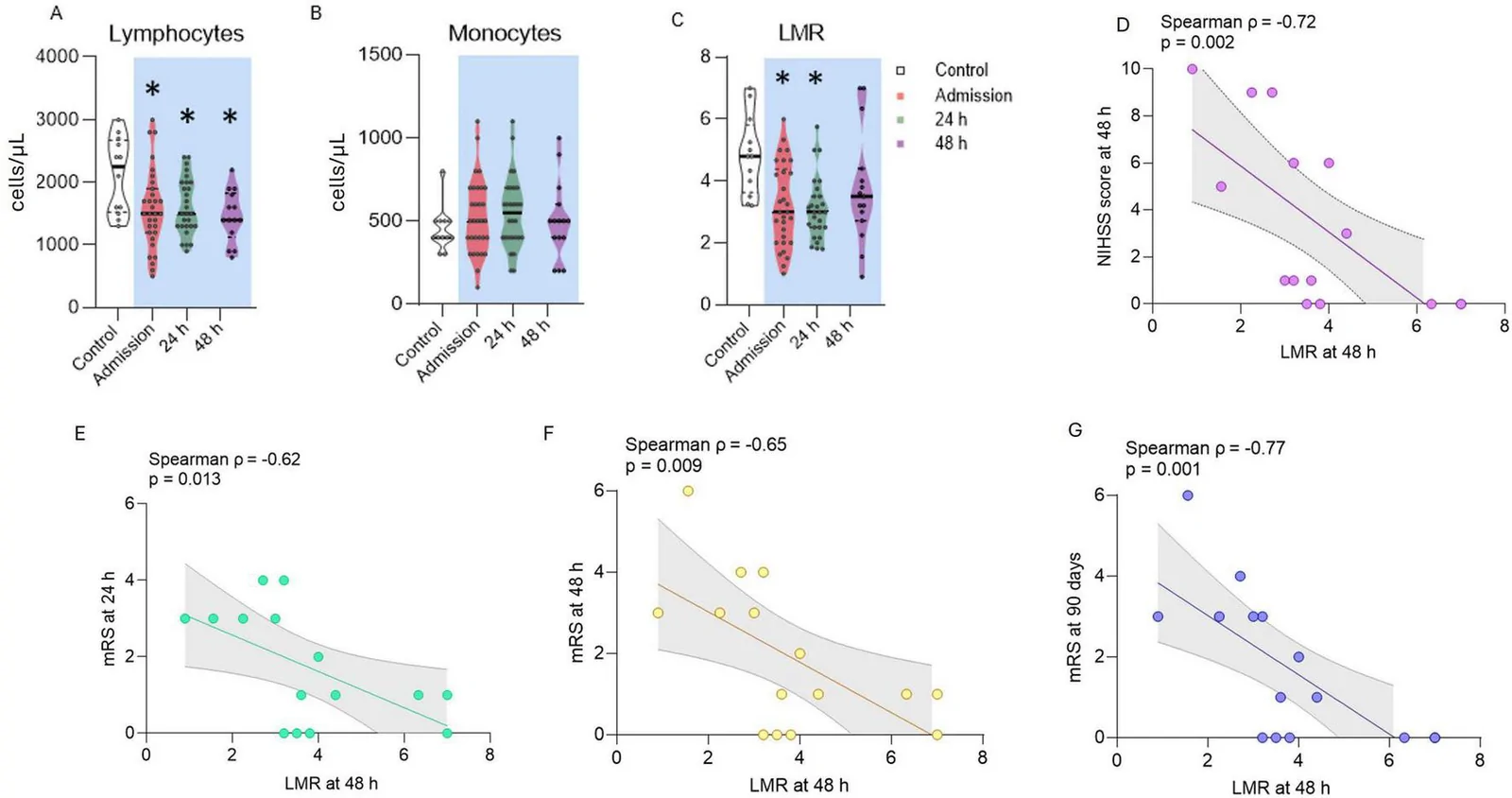
Changes in lymphocytes and monocytes in IS, and LMR correlations with functional outcome. **(A)** Lymphocyte counts vs. controls: admission *p* = 0.026, 24 h *p* = 0.012, 48 h *p* = 0.035. **(B)** Total monocyte counts: no differences vs. controls at any timepoint, nor across timepoints (Kruskal-Wallis *p* = 0.732). **(C)** LMR vs. controls: admission *p* = 0.004, 24 h *p* < 0.001, 48 h *p* = 0.99. **(D–G)** LMR at 48 h inversely correlated with NIHSS at 48 h (ρ = -0.72, *p* = 0.002), mRS at 24 h (ρ = -0.62, *p* = 0.013), mRS at 48 h (ρ = -0.65, *p* = 0.009), and mRS at 90 days (ρ = -0.77, *p* = 0.001). N. Admission (*n* = 31); 24 h (*n* = 28); 48 h (*n* = 15); controls (*n* = 12). *p < 0.05 versus controls (Mann-Whitney U test).

### Lymphocyte-to-monocyte ratio during the natural course of non-reperfused ischemic stroke and its association with stroke severity and outcome

The LMR was significantly lower in IS patients than in controls (4.80; IQR 3.75–5.63) at admission (3.00; IQR 2.00–4.67; *p* = 0.004) and at 24 h (3.00; IQR 2.50–3.63; *p* < 0.001), not at 48 h (3.50; IQR 2.71–4.40; *p* = 0.99) (Figure 2C).

The LMR at 48 h showed an inverse correlation with NIHSS score at 48 h (ρ = -0.72, *p* = 0.002) (Figure 2D), with mRS at 24 h (ρ = -0.62, *p* = 0.013) (Figure 2E), at 48 h (ρ = -0.65, *p* = 0.009) (Figure 2F), and at 90 days (ρ = -0.77, *p* = 0.001) (Figure 2G).

### Intermediate monocytes and LMRint dynamics in non-reperfused ischemic stroke

IntMo counts were significantly higher in IS patients at 24 h (138.28 cells/μL; IQR 55.10–239.48; *p* = 0.04) and at 48 h (134.06 cells/μL; IQR 80.26–222.29; *p* = 0.019) compared with controls (41.29 cells/μL; IQR 32.73–50.34) (Figure 3A). Across the three timepoints, intMo counts differed significantly overall (Kruskal-Wallis *p* = 0.003), with higher values at 24 and 48 h relative to admission (42.03 cells/μL; IQR 26.19–78.86) (Figure 3A).

**FIGURE 3:**
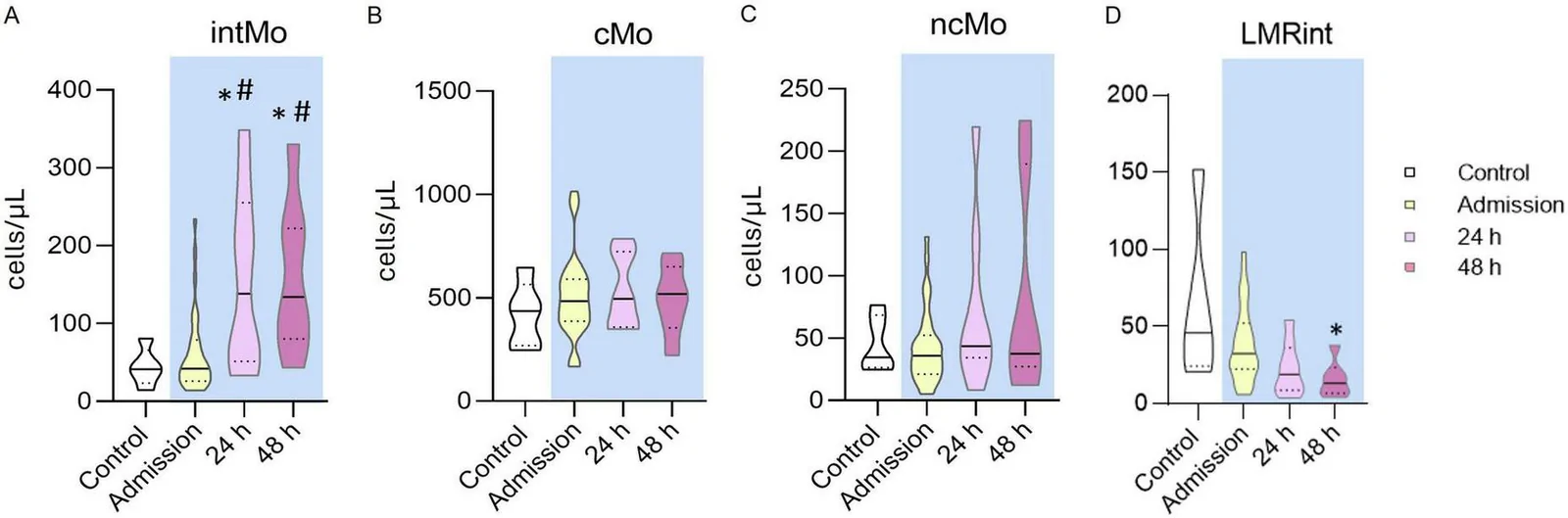
Evolution of monocyte subpopulations and LMRint in IS. **(A)** IntMo vs. controls: 24 h *p* = 0.04, 48 h *p* = 0.019; across timepoints, Kruskal-Wallis *p* = 0.003 (higher at 24–48 h vs. admission). **(B)** cMo: no differences across timepoints or vs. controls. **(C)** ncMo: no differences across timepoints or vs. controls. **(D)** LMRint vs. controls at 48 h, *p* = 0.042; across timepoints, *p* = 0.05 (progressive decline: 32.59/19.06/17.90). N. Admission (*n* = 31); 24 h (*n* = 8); 48 h (*n* = 7); controls (*n* = 5). *p < 0.05 versus controls (Mann–Whitney U test); ^#^*p* < 0.05 versus admission.

In contrast, no significant differences were observed in cMo counts (admission: 483.73 cells/μL; 24 h: 359.83 cells/μL; 48 h: 519.57 cells/μL; *p* = 0.48) or ncMo counts (admission: 36.01 cells/μL; 24 h: 44.00 cells/μL; 48 h: 55.30 cells/μL; *p* = 0.86) across time points or when compared with controls (cMo: 436.20 cells/μL; ncMo: 34.43 cells/μL) (Figures 3B,C).

A significant decrease in LMRint was observed at 48 h compared with controls (17.9 vs. 46.02; *p* = 0.042), with a progressive decline over time (32.59 at admission, 19.06 at 24 h, and 17.9 at 48 h; *p* = 0.05) (Figure 3D).

No significant associations were found between intMo counts and NIHSS or mRS scores at any time point.

Correction for multiple comparisons was applied separately within four prespecified families: a family testing LMRclas and LMRint against the four mRS assessments (24 tests), a family testing the same six biomarker variables against the four NIHSS assessments (24 tests), and two exploratory families comprising the remaining hematological and flow-cytometric variables tested against the four mRS assessments and the four NIHSS assessments, respectively (248 tests each). After FDR correction, none of the associations that were statistically significant in the unadjusted analyses retained significance within any family (all *q* > 0.05). Unadjusted and FDR-adjusted *p*-values for all analyses are provided in Supplementary Tables 2, 3.

Within the exploratory families, a number of additional correlations reached nominal significance in the unadjusted analyses; none retained significance after FDR correction, and they are not discussed further, as they fall outside the prespecified focus of the present study.

In the ordinal regression models for 90-day mRS, baseline NIHSS remained significantly associated with functional outcome (LMRclas model: adjusted OR 1.54, 95% CI 1.16–2.05, *p* = 0.003; LMRint model: adjusted OR 1.50, 95% CI 1.14–1.96, *p* = 0.003), as did age (adjusted OR 1.15, 95% CI 1.03–1.27, *p* = 0.009, and adjusted OR 1.11, 95% CI 1.02–1.20, *p* = 0.011, respectively).

Male sex was associated with lower odds of a worse 90-day mRS in the LMRclas model (adjusted OR 0.09, 95% CI 0.01–0.97, *p* = 0.047), with a similar but non-significant estimate in the LMRint model (adjusted OR 0.10, 95% CI 0.01–1.07, *p* = 0.057); the very wide confidence intervals and the small number of women in the cohort (*n* = 6) warrant caution in interpreting this association.

Neither LMRclas (adjusted OR 1.43, 95% CI 0.72–2.82, *p* = 0.307) nor LMRint (adjusted OR 0.99, 95% CI 0.96–1.03, *p* = 0.606) remained independently associated with 90-day mRS after adjustment. Similarly, in the linear regression model for 90-day NIHSS, baseline NIHSS remained significantly associated with outcome (β = 0.35, 95% CI 0.13–0.57, *p* = 0.003), whereas LMRclas did not (β = -0.09, 95% CI -0.55 to 0.38, *p* = 0.704).

The same pattern was observed in the corresponding model for LMRint, in which baseline NIHSS remained associated with 90-day NIHSS (β = 0.35, 95% CI 0.13–0.57, *p* = 0.003) whereas LMRint did not (β = 0.01, 95% CI -0.02 to 0.04, *p* = 0.541); this model explained 39.0% of the variance [adjusted *R*^2^ = 0.296; *F*(4,26) = 4.16, *p* = 0.010].

### Classical monocytes and LMRclas associations with clinical outcomes in non-reperfused ischemic stroke

A significant association was found between cMo counts at 48 h and NIHSS scores at 90 days (ρ = 0.84, *p* = 0.019) (Figure 4A).

**FIGURE 4:**
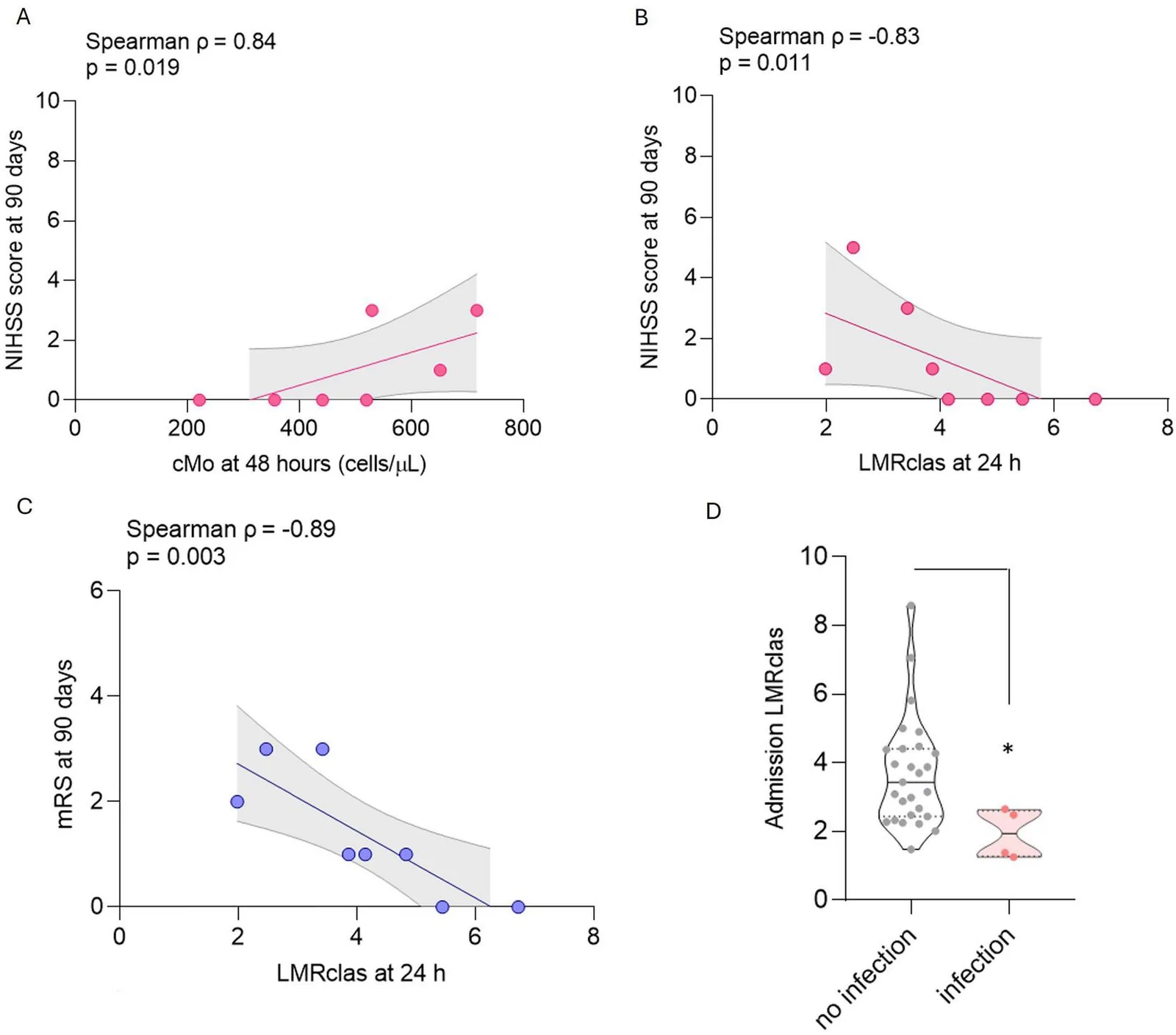
Association of cMo and LMRclas with functional outcome and infection risk in IS. **(A)** cMo at 48 h vs. NIHSS at 90 days: ρ = 0.84, *p* = 0.019. **(B)** LMRclas at 24 h vs. NIHSS at 90 days: ρ = -0.83, *p* = 0.011. **(C)** LMRclas at 24 h vs. mRS at 90 days: ρ = -0.89, *p* = 0.003. **(D)** Admission LMRclas vs. SAI: OR 0.13 (95% CI 0.02–0.99), *p* = 0.049, 4 events. N. **(A)** *n* = 7; **(B,C)**, *n* = 8 (paired flow-cytometry beyond admission); **(D)** *n* = 31 (4 infection events). *p < 0.05 for the comparison between patients with and without stroke-associated infection.

LMRclas at 24 h showed an inverse correlation with NIHSS score at 90 days (ρ = -0.83, *p* = 0.011) (Figure 4B) and with mRS score at 90 days (ρ = -0.89, *p* = 0.003), such that lower LMRclas at 24 h was associated with poorer clinical and functional outcome (Figure 4C).

Lower admission LMRclas was also associated with a higher likelihood of developing SAI during hospitalization (OR 0.13; 95% CI 0.02–0.99; *p* = 0.049) (Figure 4D).

This association should be interpreted with caution given the very small number of infection events (*n* = 4). No additional associations were identified between other monocyte subpopulations or their derived ratios and infection risk during admission (data not shown).

Receiver operating characteristic (ROC) curve analyses were performed for diagnostic discrimination between IS patients and controls, and for prognostic discrimination of poor functional outcome (mRS ≥ 3) at 90 days (Supplementary Table 4).

For diagnostic discrimination (*n* = 43: 31 patients and 12 controls with available complete blood counts), total LMR showed an AUC of 0.78 (95% CI 0.64–0.92); the Youden-optimal cut-off was 3.10, with 51.6% sensitivity and 100% specificity. Among the subset with paired flow-cytometry data (*n* = 36: 31 patients and 5 controls), LMRclas showed an AUC of 0.77 (95% CI 0.59–0.95; cut-off 3.27, sensitivity 54.8%, specificity 100%), and LMRint showed an AUC of 0.65 (95% CI 0.36–0.93; cut-off 45.75, sensitivity 71.0%, specificity 60.0%). Given the small number of controls with paired flow-cytometry data, these estimates are reported descriptively and should not be interpreted as evidence of diagnostic utility.

For prognostic discrimination of poor functional outcome at 90 days (mRS ≥ 3; *n* = 31, 9 events), total LMR showed an AUC of 0.60 (95% CI 0.38–0.83; cut-off 3.37, sensitivity 77.8%, specificity 50.0%). LMRclas showed an AUC of 0.75 (95% CI 0.53–0.97; cut-off 2.26, sensitivity 55.6%, specificity 95.5%), and LMRint showed an AUC of 0.52 (95% CI 0.29–0.75; cut-off 24.55, sensitivity 44.4%, specificity 72.7%). All three analyses were performed on the same 31 patients (22 without and 9 with a poor outcome). Given the limited number of outcome events, these prognostic estimates, particularly for LMRclas, should be interpreted with caution and regarded as exploratory. The complete output of the four adjusted models, including coefficients and 95% confidence intervals, is provided in Supplementary Table 5.

## Discussion

In this exploratory study, we characterized the dynamics of monocyte subpopulations and their lymphocyte-derived ratios during the natural course of non-reperfused IS in humans, introducing the novel concept of monocyte subpopulation ratios such as LMRclas and LMRint. Our main findings suggest that intMo may be mobilized in peripheral blood during the natural course of IS, and that cMo and its derived ratio (LMRclas) may be associated with IS outcome, SAI, and functional outcome. Our results also show an association of LMR with clinical severity and functional outcome in non-treated patients.

Acute lymphopenia was observed in our cohort, consistent with previous reports describing this phenomenon as part of the pathophysiology of IS and associating it with stroke-induced immunosuppression (Faura et al., 2021); however, total monocyte counts did not differ significantly across the three timepoints (admission, *n* = 31; 24 h, *n* = 28; 48 h, *n* = 15; *p* = 0.732), nor compared with controls (*n* = 12) at any individual timepoint.

These convergences and divergences should be read in the light of how the three cohorts were assembled. Urra et al. described monocyte subset dynamics in a single group of 46 patients combining ischemic and hemorrhagic stroke, so that the trajectory reported there reflects both pathologies together rather than cerebral ischemia in isolation (Urra et al., 2009b). Kaito et al. studied an exclusively ischemic cohort, but the comparison group consisted of 24 patients admitted with other neurological conditions, in whom an independent inflammatory contribution cannot be excluded (Kaito et al., 2013). The present study was restricted to IS, excluded patients who received thrombolysis or thrombectomy, excluded concurrent inflammatory, infectious, and neoplastic disease, and used a comparison group free of acute neurological illness, although this group was not matched for age, a difference considered in the Limitations section below. Within these constraints, the temporal profile described here can be referred to the untreated ischemic process itself with fewer competing explanations than in more heterogeneous cohorts. Comparison across studies is further limited by the nature of the available evidence: the cohorts reported to date are small and exploratory, and no effect estimate for monocyte subset dynamics in IS has yet been confirmed in an adequately powered sample.

A further distinction is clinical rather than methodological: 67.7% of patients presented with MIS (NIHSS < 5), a group that remains outside reperfusion protocols in a substantial proportion of cases and in whom the natural course of the monocyte response has been comparatively little described, a difference in clinical severity that has itself been linked to the magnitude of the post-stroke increase in monocytes (Liberale et al., 2017; Bolayir et al., 2018; Gill et al., 2018; Dong et al., 2020). Whereas earlier work characterized monocyte subsets as proportions or absolute counts, we additionally examined subset-specific ratios (LMRclas and LMRint) alongside the total LMR. These are proposed as an exploratory line of inquiry rather than as established indices. Whether partitioning the LMR by monocyte subset offers any advantage over the total ratio is a question that could not previously be posed in this population, and one that the present data are intended to make testable.

Nevertheless, we observed significant differences in LMR values compared with healthy controls. LMR at 48 h showed an inverse association with IS severity (assessed by the NIHSS at 48 h) and with functional outcome at 24 h, 48 h and 90 days (assessed by the mRS).

These findings are consistent with previous studies (Ren et al., 2017a; Park et al., 2018), which have also reported an association between lower LMR and poorer functional outcomes in IS.

We found a selective increase in circulating intMo during the acute phase of IS, as evidenced by the flow-cytometry subsample (*n* = 8 at 24 h and *n* = 7 at 48 h, versus *n* = 31 at admission and *n* = 5 in controls). IntMo counts were significantly higher at 24 and 48 h compared with admission and were also significantly higher than those observed in healthy controls at these timepoints.

In contrast, no significant changes were observed in cMo and ncMo subsets. This pattern is consistent with previous studies describing changes in monocyte subset distribution during the acute phase of IS (Urra et al., 2009b; Kaito et al., 2013; Grosse et al., 2016).

This selective increase in intMo was accompanied by a decrease in LMRint at 48 h. Similar changes in intMo-related profiles have been described in other acute vascular conditions, including myocardial infarction and heart failure (Ruder et al., 2023). Together, these findings suggest that changes in circulating intMo and LMRint may form part of the acute immune response following cerebral ischemia.

No statistically significant associations were found between intMo counts or LMRint and the NIHSS or mRS scales in our cohort.

Alterations within the bone marrow niche have been linked to vascular remodeling and IS outcomes in other contexts (Rohde et al., 2021; Endres et al., 2022); whether such changes contribute to the intMo dynamics observed here cannot be determined from our data. We therefore propose that intMo may reflect the individual immune response to disease, potentially influenced by baseline inflammatory status, vascular risk factors (Rogacev et al., 2012), and comorbid atherosclerotic disease (Wildgruber et al., 2016). Whether intMo represent a functionally distinct population with a specific role in IS, within the broader context of cardiovascular disease as a chronic inflammatory process, remains an open question for future research (Ruder et al., 2023).

Our study also found a significant association between cMo counts at 48 h and NIHSS scores at 90 days, suggesting a relationship between cMo levels and IS severity. Additionally, LMRclas at 24 h showed an inverse association with IS severity and functional outcome at 90 days. Both associations, however, derive from the small subgroup with paired flow-cytometry sampling beyond admission (*n* = 8 at 24 h and *n* = 7 at 48 h), in which only very large effects were detectable (Supplementary Table 1). This concern is particularly acute for the cMo-NIHSS90 correlation, as NIHSS at 90 days was highly concentrated at 0 in this subgroup (4 of 7 patients), further limiting the variability available for correlation analysis.

The magnitude of these coefficients should therefore not be read at face value: they indicate the direction of a relationship worth testing, not its size.

Higher cMo counts have previously been associated with poorer outcomes, mortality, and clinical worsening (Urra et al., 2009a,2009b). Translational studies have implicated the murine cMo equivalent (Ly6Chigh CCR2+) as an important monocyte effector population within the brain. The expansion of circulating cMo in the blood may reflect enhanced release of these cells from hematopoietic organs and may be consistent with their recruitment to the brain, although this mechanism cannot be established from our study (Stephens et al., 2023).

Finally, we found an association between admission LMRclas and the occurrence of SAI during hospitalization. This finding should be interpreted with particular caution, as it rests on a very small number of infection events. While biologically plausible given the established role of monocytic HLA-DR dysfunction in post-stroke infection susceptibility, this association is best regarded as hypothesis-generating rather than confirmatory. To date, few studies have specifically addressed the role of monocyte subpopulations in SAI. Monocytic HLA-DR expression is a well-established and sensitive marker of immune dysfunction in septic and critically ill patients (Lekkou et al., 2004; Monneret et al., 2006), and it is primarily altered within the cMo subset. Harms and colleagues reported that low monocytic HLA-DR levels at day 1 after IS are associated with an increased occurrence of post-stroke infection; however, they did not stratify their analysis by monocyte subpopulations (Harms et al., 2008). Further research is needed to clarify the role of cMo in the very early phases after IS and to better understand their relationship with SAI susceptibility.

Among the strengths of this study is the homogeneity of the sample, achieved through rigorous inclusion criteria designed to characterize the immune response associated with the natural course of cerebral ischemia. However, the exclusion of reperfusion therapies also limits the generalizability of our findings, which should be interpreted in the context of non-reperfused IS. Importantly, intravenous thrombolysis and mechanical thrombectomy may themselves modify peripheral immune responses.

Recombinant tPA induces neutrophil degranulation and matrix metalloproteinase release within hours of administration (Cuadrado et al., 2008), while endovascular recanalization may trigger ischemia–reperfusion injury and platelet-driven leukocyte accumulation, contributing to thromboinflammatory mechanisms that are not present to the same extent in non-reperfused ischemia (Stoll and Nieswandt, 2019; Stoll et al., 2024). Peripheral molecular immunovascular activation has also been documented in the hours following mechanical thrombectomy (Rapido et al., 2026).

In addition, collecting samples at the same time of day minimized the potential influence of circadian variation on the cell populations analyzed. The strict selection criteria further allowed us to exclude or control factors potentially related to the immune response, such as concomitant inflammatory, infectious, or neoplastic conditions and treatments that could interfere with the parameters studied.

This study has several limitations, including the small sample size, particularly for FC at 24 and 48 h, and the lack of age-matched controls (median 77 vs. 52.5 years).

As noted in Methods, retrospective power calculations were not performed given their circularity with the obtained *p*-values (Hoenig and Heisey, 2001); instead, we performed a sensitivity analysis to quantify the statistical constraints of the available sample (see Methods and Supplementary Table 1 for full values by timepoint). The results confirm that moderate effect sizes could not be reliably detected at later timepoints, particularly in the FC subanalyses.

Two opposing sources of inferential error apply to this dataset. On one hand, the large number of unadjusted comparisons performed inflates the risk of type I error, which is precisely what the FDR analysis was designed to quantify. On the other hand, the small sample size means that the correction itself substantially increases the risk of type II error (Rothman, 1990; Bender and Lange, 2001). Several associations did not retain significance after FDR adjustment, an expected consequence of applying multiplicity correction to a small exploratory dataset. These associations are therefore presented as hypothesis-generating observations, consistent in direction and magnitude with prior evidence, rather than as confirmed findings.

The multivariable models warrant the same caution. In the four parsimonious models adjusting for age, sex, and baseline NIHSS, neither LMRclas nor LMRint remained independently associated with 90-day functional or neurological outcome, whereas baseline NIHSS itself remained robustly associated with outcome. This pattern suggests that the unadjusted associations of LMRclas and LMRint with outcome may partly reflect the underlying severity of the IS rather than an independent contribution of these ratios. Given the limited number of clinical events, more comprehensive adjustment for vascular risk factors and IS subtype was not attempted, as this risked producing unstable estimates (Peduzzi et al., 1996). These findings do not negate the unadjusted associations reported above, but indicate that the independent prognostic contribution of LMRclas and LMRint, beyond IS severity itself, remains to be established in larger cohorts.

The discriminative performance of the LMR-based indices in this cohort, expressed as areas under the ROC curve, was modest. This is broadly consistent with the existing literature, in which the discriminative performance of LMR for IS outcomes has been variable, with reported AUC values ranging from modest to relatively good across individual studies (Gong et al., 2021; Wang et al., 2024). A recent meta-analysis of six retrospective studies (1,225 patients) found that lower LMR was associated with poor 3-month functional outcome (pooled OR 0.63; 95% CI 0.49–0.80), but did not report a pooled AUC, underscoring the uncertainty around the ratio’s discriminative value at the individual-patient level (Tian et al., 2025).

Across studies, reported LMR cut-offs have been highly heterogeneous (Ren et al., 2017b; Pinčáková et al., 2022), likely reflecting differences in population, sampling timepoint, and hematology analyzers. None of these cut-offs has been externally validated.

To our knowledge, no previous study has reported ROC performance for monocyte subset counts or subset-derived ratios such as LMRclas and LMRint in IS; the seminal studies establishing their prognostic relevance (Urra et al., 2009b; Kaito et al., 2013) did not include ROC analyses. Our subset-derived ratios may therefore reflect distinct immunological processes not captured by the conventional whole-monocyte LMR. Given the small sample size and limited number of events, these estimates should be regarded as exploratory and require confirmation in larger, externally validated cohorts.

Although Youden-optimal cut-off, sensitivity, and specificity values are reported for completeness (Supplementary Table 4), including for the prognostic use of LMRclas and LMRint, these should not be interpreted as validated clinical thresholds: with only 9 outcome events, the resulting estimates carry considerable uncertainty and are likely to be unstable if recalculated in an independent sample.

Another important limitation is the age difference between patients with IS and healthy controls (median age, 77 vs. 52.5 years, respectively), which may introduce age-related confounding in the case-control comparisons. Within our IS cohort (age range, 47–94 years; *n* = 31), we found no significant correlation between age and the proportions of cMo, intMo or ncMo at baseline (Spearman’s ρ = 0.23, -0.07, and -0.23, respectively; all *p* > 0.2).

This analysis is necessarily indirect, however, as it examines age–proportion relationships within the restricted age range of the IS cohort and does not, by itself, exclude age-related confounding in the case-control comparison, particularly given the limited overlap between the age distributions of patients (47–94 years) and controls (40–61 years). A direct test across the combined age range was precluded by the small number of controls with paired FC data (*n* = 5).

The available evidence regarding the effect of aging on monocyte subset distribution is not entirely consistent. Some studies have reported that the relative proportions of cMo, intMo, and ncMo remain relatively stable across adulthood (Reitsema et al., 2024; Tampé et al., 2024), whereas others have described an age-related shift towards CD16-expressing subsets (Hearps et al., 2012; Cao et al., 2022; Snodgrass et al., 2022). Importantly, when absolute counts are considered, age-related changes appear to be mainly attributable to an increase in ncMo, while cMo counts remain relatively stable (Seidler et al., 2010). Thus, although an age-related contribution cannot be entirely ruled out, the age mismatch is less likely to account for our principal findings involving cMo and intMo.

A related but distinct concern applies to the LMR itself, which may be more susceptible to age-related confounding than the subset proportions discussed above. Lymphocyte counts decline with age, particularly involving CD8+ T cells and B cells after the fifth decade (Jentsch-Ullrich et al., 2005), and lower LMR values have been reported in older healthy populations (Moosazadeh et al., 2019; Wang et al., 2021). Therefore, the lower LMR observed in patients compared with controls may partly reflect the age difference between groups and should be interpreted with appropriate caution.

Importantly, this limitation applies specifically to the case-control comparisons. The longitudinal analyses within the IS cohort, including the temporal increase in intermediate monocytes, the progressive decline in LMRint, and the associations of LMR, cMo, and LMRclas with NIHSS, mRS, and SAI, are based on comparisons that do not involve the control group, and are therefore not affected by the age difference between patients and controls.

Larger studies with appropriately age-matched controls will be necessary to definitively distinguish stroke-related alterations from age-related differences in immune cell profiles.

Read together, the findings reported here are exploratory, and their value lies less in any individual association than in what the study establishes for the work that follows. The exclusion of reperfusion therapy allowed monocyte subset dynamics to be observed without the immune perturbation introduced by thrombolysis or thrombectomy, and the strict inclusion criteria excluded concurrent inflammatory, infectious, and neoplastic conditions; the temporal pattern described here can therefore be attributed to the ischemic process itself with fewer competing explanations than in more heterogeneous cohorts. The results are also quantitative in a way that is directly usable: because no prior estimates existed for LMRclas or LMRint in IS, an adequately powered study of these ratios could not previously be designed. The effect estimates and confidence intervals reported here, together with the sensitivity analysis in Supplementary Table 1, provide that basis. Seen in this light, the associations that did not survive correction for multiple comparisons, and the absence of an independent contribution of either ratio beyond IS severity, are themselves informative: they suggest that any true effect is unlikely to be as large as the point estimates from the smallest subgroups would imply, and they inform the magnitude for which a confirmatory study should be powered.

## Conclusion

This exploratory study provides, to our knowledge, the first characterization of monocyte subpopulation ratios, LMRclas and LMRint, and of their dynamics during the natural course of non-reperfused IS in humans. The principal findings are a selective increase in circulating intMo during the acute phase, a progressive decline in LMRint, and associations of LMRclas at 24 h with functional outcome at 90 days and of LMRclas at admission with the occurrence of SAI during hospitalization. These associations were observed in unadjusted analyses and did not retain statistical significance after correction for multiple comparisons; in models adjusted for age, sex, and baseline NIHSS, neither ratio remained independently associated with 90-day outcome, whereas baseline IS severity did. The present results are therefore hypothesis-generating and suggest that monocyte subset dynamics and their derived ratios may reflect aspects of the immune response relevant to the clinical course of IS, warranting further investigation of LMRclas and LMRint as candidate parameters for patient characterization. Validation in larger, independent, prospective cohorts, adequately powered and including patients receiving reperfusion therapies, is required. Future work should combine subset quantification with functional markers of monocyte competence and extend sampling beyond 48 h to capture the subacute phase in which reparative monocyte functions predominate.

## Data Availability

De-identified data supporting the conclusions of this article may be made available to qualified researchers for a specified research purpose, subject to approval by the corresponding Clinical Research Ethics Committee and to a data transfer agreement. Aggregate data supporting the conclusions of this article are provided in Supplementary Tables 1–5. Requests to access the datasets should be directed to the corresponding authors.

## Footnotes

1
www.euroflow.org
