# Supplementary material for: Classical and intermediate monocytes and their ratios in the natural immune course of ischemic stroke
Source: Front Neurosci. 2026 Sep 30;20:1889912. doi: 10.3389/fnins.2026.1889912 (PMC13661920; doi:10.3389/fnins.2026.1889912)
Supplement: Supplementary file 1 [file Table_1.docx]

**Supplementary Table 1. Sensitivity analysis of statistical power**

| **Timepoint** | **n** | **Analysis / sample** | **Minimum detectable correlation \|r\|** | **Minimum detectable Cohen's d** |
| --- | --- | --- | --- | --- |
| Admission (baseline) | 31 | Full cohort | ≥ 0.485 | ≥ 0.88 |
| 24 hours | 8 | Flow cytometry subset | ≥ 0.849 | ≥ 1.69 |
| 48 hours | 7 | Flow cytometry subset | ≥ 0.886 | ≥ 1.76 |
| 48 hours | 15 | Full longitudinal sample (CBC) | ≥ 0.669 | ≥ 0.91 |

*Minimum effect size detectable with 80% power at a two-sided α = 0.05, estimated separately for correlational analyses (expressed as minimum detectable |r|) and for between-group comparisons (expressed as Cohen's d), at each study timepoint. At 48 hours, estimates are shown separately for the flow-cytometry subset (n = 7) and for the full longitudinal sample with complete blood counts (n = 15). Retrospective (post-hoc) power calculations were not performed, as this approach is mathematically circular with the obtained p-values and does not provide additional inferential information (Hoenig and Heisey, 2001).*

*Note on notation. Power estimates for correlational analyses are conventionally derived from the Pearson correlation coefficient, whose sampling distribution under the null hypothesis is analytically defined; the minimum detectable effect is therefore expressed as |r|. As the correlation analyses reported in this study used Spearman's rank correlation (ρ), these values should be interpreted as approximate lower bounds on the minimum detectable |ρ|. Under bivariate normality the asymptotic relative efficiency of Spearman's ρ relative to Pearson's r is approximately 0.91, so the minimum detectable |ρ| is expected to be marginally larger than the |r| values shown.*
