# Supplementary material for: Classical and intermediate monocytes and their ratios in the natural immune course of ischemic stroke
Source: Front Neurosci. 2026 Sep 30;20:1889912. doi: 10.3389/fnins.2026.1889912 (PMC13661920; doi:10.3389/fnins.2026.1889912)
Supplement: Supplementary file 3 [file Table_3.docx]

**Supplementary Table 3. Correlations between hematological and flow-cytometric variables and mRS scores: unadjusted (Spearman) and FDR-adjusted.**

| **Variable** | **mRS admission** | | | | | **mRS 24h** | | | | | **mRS 48h** | | | | | **mRS 90 days** | | | | |
| --- | --- | --- | --- | --- | --- | --- | --- | --- | --- | --- | --- | --- | --- | --- | --- | --- | --- | --- | --- | --- |
|  | **ρ** | **p-value** | **p-value Adj** | **Significant after FDR (q<0.05)** | **N** | **ρ** | **p-value** | **p-value Adj** | **Significant after FDR (q<0.05)** | **N** | **ρ** | **p-value** | **p-value Adj** | **Significant after FDR (q<0.05)** | **N** | **ρ** | **p-value** | **p-value Adj** | **Significant after FDR (q<0.05)** | **N** |
| ***Primary family (LMRclas and LMRint)*** | | | | | | | | | | | | | | | | | | | | |
| LMRclas_adm | 0.0455 | 0.8080 | 0.9149 | No | 31 | −0.1034 | 0.5797 | 0.8076 | No | 31 | −0.1545 | 0.4067 | 0.8076 | No | 31 | −0.3323 | 0.0677 | 0.7673 | No | 31 |
| LMRclas_24h | −0.1596 | 0.7058 | 0.8470 | No | 8 | −0.2718 | 0.5149 | 0.8076 | No | 8 | −0.2946 | 0.4787 | 0.8076 | No | 8 | −0.8895 | 0.0031 | 0.0744 | No | 8 |
| LMRclas_48h | −0.0371 | 0.9371 | 0.9371 | No | 7 | 0.2390 | 0.6057 | 0.8076 | No | 7 | 0.2390 | 0.6057 | 0.8076 | No | 7 | −0.5052 | 0.2474 | 0.8076 | No | 7 |
| LMRint_adm | 0.2305 | 0.2123 | 0.8076 | No | 31 | 0.3044 | 0.0959 | 0.7673 | No | 31 | 0.2441 | 0.1858 | 0.8076 | No | 31 | −0.0290 | 0.8768 | 0.9149 | No | 31 |
| LMRint_24h | 0.1596 | 0.7058 | 0.8470 | No | 8 | 0.4324 | 0.2846 | 0.8076 | No | 8 | 0.4419 | 0.2730 | 0.8076 | No | 8 | −0.0865 | 0.8387 | 0.9149 | No | 8 |
| LMRint_48h | −0.2965 | 0.5185 | 0.8076 | No | 7 | 0.2789 | 0.5447 | 0.8076 | No | 7 | 0.2789 | 0.5447 | 0.8076 | No | 7 | −0.3555 | 0.4338 | 0.8076 | No | 7 |
| ***Exploratory family*** | | | | | | | | | | | | | | | | | | | | |
| Basophils_adm | −0.3930 | 0.0288 | 0.5381 | No | 31 | −0.2981 | 0.1034 | 0.5533 | No | 31 | −0.3066 | 0.0934 | 0.5533 | No | 31 | −0.3267 | 0.0728 | 0.5533 | No | 31 |
| Eosinophils_adm | −0.1717 | 0.3557 | 0.7115 | No | 31 | −0.0896 | 0.6315 | 0.8608 | No | 31 | −0.1474 | 0.4288 | 0.7650 | No | 31 | −0.0812 | 0.6641 | 0.8753 | No | 31 |
| Lymphocytes_adm | 0.1050 | 0.5739 | 0.8207 | No | 31 | −0.2105 | 0.2557 | 0.6284 | No | 31 | −0.2596 | 0.1585 | 0.5615 | No | 31 | −0.2916 | 0.1115 | 0.5533 | No | 31 |
| Monocytes_adm | 0.0036 | 0.9848 | 1.0000 | No | 31 | −0.1445 | 0.4381 | 0.7706 | No | 31 | −0.1294 | 0.4877 | 0.7723 | No | 31 | −0.0173 | 0.9265 | 0.9910 | No | 31 |
| Neutrophils_adm | 0.2815 | 0.1249 | 0.5533 | No | 31 | 0.1672 | 0.3687 | 0.7143 | No | 31 | 0.1984 | 0.2848 | 0.6576 | No | 31 | 0.2448 | 0.1845 | 0.5734 | No | 31 |
| Basophils_24h | 0.0807 | 0.6831 | 0.8778 | No | 28 | −0.1882 | 0.3375 | 0.6941 | No | 28 | −0.1878 | 0.3386 | 0.6941 | No | 28 | −0.1345 | 0.4950 | 0.7723 | No | 28 |
| Basophils_48h | −0.3436 | 0.2099 | 0.5983 | No | 15 | −0.4961 | 0.0600 | 0.5533 | No | 15 | −0.4933 | 0.0617 | 0.5533 | No | 15 | −0.5436 | 0.0362 | 0.5381 | No | 15 |
| ENR_24h | −0.3571 | 0.0621 | 0.5533 | No | 28 | −0.3705 | 0.0523 | 0.5533 | No | 28 | −0.4230 | 0.0249 | 0.5381 | No | 28 | −0.2037 | 0.2984 | 0.6633 | No | 28 |
| ENR_48h | −0.3908 | 0.1497 | 0.5615 | No | 15 | −0.3680 | 0.1772 | 0.5633 | No | 15 | −0.4406 | 0.1002 | 0.5533 | No | 15 | −0.3592 | 0.1885 | 0.5751 | No | 15 |
| ENR_adm | −0.2844 | 0.1210 | 0.5533 | No | 31 | −0.1703 | 0.3596 | 0.7133 | No | 31 | −0.2194 | 0.2356 | 0.6284 | No | 31 | −0.1223 | 0.5123 | 0.7776 | No | 31 |
| Eosinophils_24h | −0.3247 | 0.0918 | 0.5533 | No | 28 | −0.3087 | 0.1099 | 0.5533 | No | 28 | −0.3691 | 0.0532 | 0.5533 | No | 28 | −0.1328 | 0.5005 | 0.7723 | No | 28 |
| Eosinophils_48h | −0.3580 | 0.1901 | 0.5751 | No | 15 | −0.1964 | 0.4829 | 0.7723 | No | 15 | −0.2841 | 0.3048 | 0.6633 | No | 15 | −0.2212 | 0.4282 | 0.7650 | No | 15 |
| LMR_24h | −0.0598 | 0.7623 | 0.8990 | No | 28 | −0.1684 | 0.3917 | 0.7275 | No | 28 | −0.1925 | 0.3265 | 0.6927 | No | 28 | −0.2991 | 0.1221 | 0.5533 | No | 28 |
| LMR_48h | −0.4293 | 0.1103 | 0.5533 | No | 15 | −0.6240 | 0.0129 | 0.4578 | No | 15 | −0.6497 | 0.0087 | 0.3616 | No | 15 | −0.7712 | 0.0008 | 0.0969 | No | 15 |
| LMR_adm | 0.1804 | 0.3316 | 0.6927 | No | 31 | 0.0128 | 0.9456 | 0.9910 | No | 31 | −0.0343 | 0.8547 | 0.9548 | No | 31 | −0.1925 | 0.2995 | 0.6633 | No | 31 |
| LMRnc_adm | −0.1502 | 0.4201 | 0.7649 | No | 31 | 0.1338 | 0.4729 | 0.7723 | No | 31 | 0.1248 | 0.5037 | 0.7723 | No | 31 | −0.1088 | 0.5600 | 0.8172 | No | 31 |
| LMRnc_24h | 0.0614 | 0.8852 | 0.9800 | No | 8 | 0.2965 | 0.4758 | 0.7723 | No | 8 | 0.3560 | 0.3868 | 0.7275 | No | 8 | −0.0988 | 0.8159 | 0.9368 | No | 8 |
| LMRnc_48h | −0.0741 | 0.8745 | 0.9725 | No | 7 | 0.5777 | 0.1744 | 0.5633 | No | 7 | 0.5777 | 0.1744 | 0.5633 | No | 7 | −0.2058 | 0.6579 | 0.8753 | No | 7 |
| Lymphocytes_24h | 0.2258 | 0.2479 | 0.6284 | No | 28 | −0.1150 | 0.5602 | 0.8172 | No | 28 | −0.0975 | 0.6215 | 0.8608 | No | 28 | −0.1414 | 0.4729 | 0.7723 | No | 28 |
| Lymphocytes_48h | −0.2386 | 0.3919 | 0.7275 | No | 15 | −0.2380 | 0.3931 | 0.7275 | No | 15 | −0.2505 | 0.3679 | 0.7143 | No | 15 | −0.3036 | 0.2713 | 0.6468 | No | 15 |
| Lymphocytes_abs_adm | 0.1418 | 0.4466 | 0.7713 | No | 31 | −0.0177 | 0.9248 | 0.9910 | No | 31 | −0.0786 | 0.6741 | 0.8753 | No | 31 | −0.2112 | 0.2540 | 0.6284 | No | 31 |
| Lymphocytes_abs_24h | −0.1605 | 0.7041 | 0.8909 | No | 8 | 0.0373 | 0.9302 | 0.9910 | No | 8 | 0.0185 | 0.9653 | 1.0000 | No | 8 | −0.5655 | 0.1441 | 0.5615 | No | 8 |
| Lymphocytes_abs_48h | −0.2224 | 0.6317 | 0.8608 | No | 7 | 0.2590 | 0.5750 | 0.8207 | No | 7 | 0.2590 | 0.5750 | 0.8207 | No | 7 | −0.5239 | 0.2274 | 0.6284 | No | 7 |
| Monocytes_24h | 0.2211 | 0.2582 | 0.6284 | No | 28 | 0.0402 | 0.8391 | 0.9447 | No | 28 | 0.0692 | 0.7263 | 0.8990 | No | 28 | 0.1358 | 0.4908 | 0.7723 | No | 28 |
| Monocytes_48h | 0.2995 | 0.2782 | 0.6508 | No | 15 | 0.3619 | 0.1850 | 0.5734 | No | 15 | 0.4142 | 0.1248 | 0.5533 | No | 15 | 0.4303 | 0.1094 | 0.5533 | No | 15 |
| Monocytes_abs_adm | 0.0827 | 0.6583 | 0.8753 | No | 31 | 0.0798 | 0.6697 | 0.8753 | No | 31 | 0.0874 | 0.6403 | 0.8677 | No | 31 | 0.0842 | 0.6526 | 0.8753 | No | 31 |
| Monocytes_abs_24h | 0.1367 | 0.7469 | 0.8990 | No | 8 | 0.1500 | 0.7229 | 0.8990 | No | 8 | 0.2050 | 0.6263 | 0.8608 | No | 8 | −0.1250 | 0.7680 | 0.8990 | No | 8 |
| Monocytes_abs_48h | 0.0566 | 0.9040 | 0.9910 | No | 7 | 0.0000 | 1.0000 | 1.0000 | No | 7 | 0.0000 | 1.0000 | 1.0000 | No | 7 | −0.5431 | 0.2078 | 0.5983 | No | 7 |
| cMo_abs_adm | 0.2751 | 0.1342 | 0.5609 | No | 31 | 0.1269 | 0.4964 | 0.7723 | No | 31 | 0.1284 | 0.4914 | 0.7723 | No | 31 | 0.1803 | 0.3318 | 0.6927 | No | 31 |
| cMo_abs_24h | −0.3069 | 0.4597 | 0.7723 | No | 8 | 0.2965 | 0.4758 | 0.7723 | No | 8 | 0.3314 | 0.4226 | 0.7649 | No | 8 | 0.7042 | 0.0512 | 0.5533 | No | 8 |
| cMo_abs_48h | 0.1482 | 0.7511 | 0.8990 | No | 7 | 0.0996 | 0.8317 | 0.9419 | No | 7 | 0.0996 | 0.8317 | 0.9419 | No | 7 | 0.5426 | 0.2082 | 0.5983 | No | 7 |
| Monocytes_estimated_adm | 0.2384 | 0.1966 | 0.5873 | No | 31 | 0.0790 | 0.6729 | 0.8753 | No | 31 | 0.0805 | 0.6668 | 0.8753 | No | 31 | 0.2108 | 0.2550 | 0.6284 | No | 31 |
| Monocytes_estimated_24h | −0.2578 | 0.5376 | 0.8032 | No | 8 | −0.0124 | 0.9768 | 1.0000 | No | 8 | 0.0123 | 0.9770 | 1.0000 | No | 8 | 0.6548 | 0.0781 | 0.5533 | No | 8 |
| Monocytes_estimated_48h | 0.1482 | 0.7511 | 0.8990 | No | 7 | −0.1394 | 0.7656 | 0.8990 | No | 7 | −0.1394 | 0.7656 | 0.8990 | No | 7 | 0.6175 | 0.1395 | 0.5609 | No | 7 |
| intMo_abs_adm | −0.1006 | 0.5902 | 0.8364 | No | 31 | −0.3219 | 0.0774 | 0.5533 | No | 31 | −0.2906 | 0.1127 | 0.5533 | No | 31 | −0.0766 | 0.6822 | 0.8778 | No | 31 |
| intMo_abs_24h | −0.4542 | 0.2582 | 0.6284 | No | 8 | −0.4695 | 0.2405 | 0.6284 | No | 8 | −0.4788 | 0.2301 | 0.6284 | No | 8 | 0.0000 | 1.0000 | 1.0000 | No | 8 |
| intMo_abs_48h | 0.1482 | 0.7511 | 0.8990 | No | 7 | −0.4980 | 0.2554 | 0.6284 | No | 7 | −0.4980 | 0.2554 | 0.6284 | No | 7 | 0.1497 | 0.7487 | 0.8990 | No | 7 |
| ncMo_abs_adm | 0.2711 | 0.1402 | 0.5609 | No | 31 | −0.1414 | 0.4479 | 0.7713 | No | 31 | −0.1722 | 0.3543 | 0.7115 | No | 31 | 0.0032 | 0.9863 | 1.0000 | No | 31 |
| ncMo_abs_24h | −0.3560 | 0.3868 | 0.7275 | No | 8 | −0.3953 | 0.3324 | 0.6927 | No | 8 | −0.4542 | 0.2582 | 0.6284 | No | 8 | 0.0124 | 0.9768 | 1.0000 | No | 8 |
| ncMo_abs_48h | −0.0371 | 0.9371 | 0.9910 | No | 7 | −0.5777 | 0.1744 | 0.5633 | No | 7 | −0.5777 | 0.1744 | 0.5633 | No | 7 | 0.2994 | 0.5142 | 0.7776 | No | 7 |
| NMR_24h | 0.1789 | 0.3624 | 0.7133 | No | 28 | 0.3369 | 0.0796 | 0.5533 | No | 28 | 0.3300 | 0.0864 | 0.5533 | No | 28 | 0.1272 | 0.5190 | 0.7801 | No | 28 |
| NMR_48h | 0.0188 | 0.9470 | 0.9910 | No | 15 | 0.2084 | 0.4561 | 0.7723 | No | 15 | 0.1870 | 0.5045 | 0.7723 | No | 15 | 0.2095 | 0.4536 | 0.7723 | No | 15 |
| NMR_adm | 0.1966 | 0.2890 | 0.6576 | No | 31 | 0.2745 | 0.1351 | 0.5609 | No | 31 | 0.2892 | 0.1146 | 0.5533 | No | 31 | 0.2528 | 0.1701 | 0.5633 | No | 31 |
| Neutrophils_24h | 0.4988 | 0.0069 | 0.3422 | No | 28 | 0.4053 | 0.0324 | 0.5381 | No | 28 | 0.4287 | 0.0228 | 0.5381 | No | 28 | 0.3468 | 0.0706 | 0.5533 | No | 28 |
| Neutrophils_48h | 0.5446 | 0.0358 | 0.5381 | No | 15 | 0.6687 | 0.0064 | 0.3422 | No | 15 | 0.7136 | 0.0028 | 0.2325 | No | 15 | 0.7702 | 0.0008 | 0.0969 | No | 15 |
| cMo_prop_adm | −0.0423 | 0.8213 | 0.9386 | No | 31 | 0.2018 | 0.2763 | 0.6508 | No | 31 | 0.2078 | 0.2620 | 0.6307 | No | 31 | 0.1125 | 0.5468 | 0.8120 | No | 31 |
| cMo_prop_24h | 0.2087 | 0.6199 | 0.8608 | No | 8 | 0.5930 | 0.1213 | 0.5533 | No | 8 | 0.6015 | 0.1147 | 0.5533 | No | 8 | 0.1235 | 0.7707 | 0.8990 | No | 8 |
| cMo_prop_48h | 0.0371 | 0.9371 | 0.9910 | No | 7 | 0.5976 | 0.1565 | 0.5615 | No | 7 | 0.5976 | 0.1565 | 0.5615 | No | 7 | 0.0374 | 0.9365 | 0.9910 | No | 7 |
| intMo_prop_adm | −0.2954 | 0.1067 | 0.5533 | No | 31 | −0.3764 | 0.0369 | 0.5381 | No | 31 | −0.3412 | 0.0603 | 0.5533 | No | 31 | −0.1428 | 0.4434 | 0.7713 | No | 31 |
| intMo_prop_24h | −0.1105 | 0.7945 | 0.9208 | No | 8 | −0.5930 | 0.1213 | 0.5533 | No | 8 | −0.6015 | 0.1147 | 0.5533 | No | 8 | −0.2347 | 0.5758 | 0.8207 | No | 8 |
| intMo_prop_48h | −0.0371 | 0.9371 | 0.9910 | No | 7 | −0.5976 | 0.1565 | 0.5615 | No | 7 | −0.5976 | 0.1565 | 0.5615 | No | 7 | −0.0374 | 0.9365 | 0.9910 | No | 7 |
| ncMo_prop_adm | 0.1856 | 0.3174 | 0.6845 | No | 31 | −0.1733 | 0.3511 | 0.7115 | No | 31 | −0.2093 | 0.2585 | 0.6284 | No | 31 | −0.0716 | 0.7017 | 0.8909 | No | 31 |
| ncMo_prop_24h | −0.1228 | 0.7721 | 0.8990 | No | 8 | −0.3583 | 0.3835 | 0.7275 | No | 8 | −0.4297 | 0.2881 | 0.6576 | No | 8 | −0.0494 | 0.9075 | 0.9910 | No | 8 |
| ncMo_prop_48h | −0.1853 | 0.6908 | 0.8831 | No | 7 | −0.7968 | 0.0319 | 0.5381 | No | 7 | −0.7968 | 0.0319 | 0.5381 | No | 7 | 0.0936 | 0.8419 | 0.9447 | No | 7 |

*Spearman's rank correlation coefficients (ρ) between each variable and the mRS at admission, 24 hours, 48 hours, and 90 days, with unadjusted p-values, Benjamini-Hochberg adjusted p-values (p-value Adj, q), and the number of paired observations (N) for each comparison. LMRclas and LMRint at the three sampling timepoints (first six rows) constitute the mRS-primary family (6 variables × 4 outcome assessments = 24 tests), corrected independently; all remaining variables constitute the mRS-exploratory family. The Benjamini-Hochberg correction was applied across the complete exploratory family of 248 tests (62 variables × 4 outcome assessments). The eight variables not displayed here correspond to the complete blood count obtained at the 90-day follow-up visit, which lies outside the acute-phase scope of the present analyses; they were retained in the correction so that the reported q-values reflect every test performed, which is the conservative choice, as excluding them would have reduced the number of tests and therefore the adjusted p-values. The q-values shown are those obtained from the correction across the complete family. No correlation retained significance after correction in any family. Variables with the suffix “abs” are the absolute counts recorded in the flow-cytometry–paired subsample (n = 31, 8, and 7 at admission, 24 hours, and 48 hours, respectively), from which the monocyte-subset ratios were derived; the corresponding variables without this suffix are the absolute counts from the full complete blood count sample (n = 31, 28, and 15). The two therefore refer to the same analyte measured in different subsets of patients, which accounts for the differing N values. Abbreviations: adm, admission; abs, absolute count in the flow-cytometry–paired subsample; prop, proportion of total monocytes; cMo, classical monocytes; intMo, intermediate monocytes; ncMo, non-classical monocytes; LMR, lymphocyte-to-monocyte ratio; LMRclas, lymphocyte-to-classical monocyte ratio; LMRint, lymphocyte-to-intermediate monocyte ratio; LMRnc, lymphocyte-to-non-classical monocyte ratio; NMR, neutrophil-to-monocyte ratio; ENR, eosinophil-to-neutrophil ratio.*
