# Supplementary material for: Classical and intermediate monocytes and their ratios in the natural immune course of ischemic stroke
Source: Front Neurosci. 2026 Sep 30;20:1889912. doi: 10.3389/fnins.2026.1889912 (PMC13661920; doi:10.3389/fnins.2026.1889912)
Supplement: Supplementary file 4 [file Table_4.docx]

| **Ratio** | **Figure** | **Analysis** | **n** | **n, Group A*** | **n, Group B*** | **AUC (95% CI)** | **Optimal Cut-off** | **Sensitivity / Specificity** |
| --- | --- | --- | --- | --- | --- | --- | --- | --- |
| **LMR** | A | Diagnostic (IS vs control) | 43 | 12 | 31 | 0.78 (0.64–0.92) | 3.10 | 51.6% / 100.0% |
| **LMR** | D | Prognostic (mRS ≥3, 90 days) | 31 | 22 | 9 | 0.60 (0.38–0.83) | 3.37 | 77.8% / 50.0% |
| **LMRclas** | B | Diagnostic (IS vs control) | 36 | 5 | 31 | 0.77 (0.59–0.95) | 3.27 | 54.8% / 100.0% |
| **LMRclas** | E | Prognostic (mRS ≥3, 90 days) | 31 | 22 | 9 | 0.75 (0.53–0.97) | 2.26 | 55.6% / 95.5% |
| **LMRint** | C | Diagnostic (IS vs control) | 36 | 5 | 31 | 0.65 (0.36–0.93) | 45.75 | 71.0% / 60.0% |
| **LMRint** | F | Prognostic (mRS ≥3, 90 days) | 31 | 22 | 9 | 0.52 (0.29–0.75) | 24.55 | 44.4% / 72.7% |

**Supplementary Table 4. Diagnostic and prognostic performance of LMR, LMRclas and LMRint**

*95% confidence intervals were calculated using the DeLong method. Optimal cut-offs were determined using the Youden index.*

**For diagnostic comparisons, Group A = controls and Group B = IS patients. For prognostic comparisons, Group A = patients without a poor outcome (mRS <3) and Group B = patients with a poor outcome (mRS ≥3) at 90 days.*
