# Supplementary material for: Classical and intermediate monocytes and their ratios in the natural immune course of ischemic stroke
Source: Front Neurosci. 2026 Sep 30;20:1889912. doi: 10.3389/fnins.2026.1889912 (PMC13661920; doi:10.3389/fnins.2026.1889912)
Supplement: Supplementary file 5 [file Table_5.docx]

**Supplementary Table 5. Multivariable regression models for 90-day outcome**

| **Model 1 — Ordinal logistic regression: 90-day mRS (0–6) ~ LMRclas (adjusted OR)** | | |
| --- | --- | --- |
| **Variable** | **Adjusted OR (95% CI)** | **p-value** |
| **LMRclas** | **1.43 (0.72–2.82)** | **0.307** |
| Age | 1.15 (1.03–1.27) | 0.009 |
| Sex (male vs female) | 0.09 (0.01–0.97) | 0.047 |
| Baseline NIHSS | 1.54 (1.16–2.05) | 0.003 |
| **Model 2 — Ordinal logistic regression: 90-day mRS (0–6) ~ LMRint (adjusted OR)** | | |
| **Variable** | **Adjusted OR (95% CI)** | **p-value** |
| **LMRint** | **0.99 (0.96–1.03)** | **0.606** |
| Age | 1.11 (1.02–1.20) | 0.011 |
| Sex (male vs female) | 0.10 (0.01–1.07) | 0.057 |
| Baseline NIHSS | 1.50 (1.14–1.96) | 0.003 |
| **Model 3 — Linear regression: 90-day NIHSS ~ LMRclas (adjusted β)** | | |
| **Variable** | **Adjusted β (95% CI)** | **p-value** |
| **LMRclas** | **−0.09 (−0.55 to 0.38)** | **0.704** |
| Age | 0.02 (−0.04 to 0.08) | 0.566 |
| Sex (male vs female) | 0.04 (−1.66 to 1.75) | 0.957 |
| Baseline NIHSS | 0.35 (0.13 to 0.57) | 0.003 |
| **Model 4 — Linear regression: 90-day NIHSS ~ LMRint (adjusted β)** | | |
| **Variable** | **Adjusted β (95% CI)** | **p-value** |
| **LMRint** | **0.01 (−0.02 to 0.04)** | **0.541** |
| Age | 0.03 (−0.03 to 0.08) | 0.333 |
| Sex (male vs female) | 0.06 (−1.64 to 1.76) | 0.941 |
| Baseline NIHSS | 0.35 (0.13 to 0.57) | 0.003 |

*Four parsimonious models were fitted given the limited number of clinical events: two ordinal logistic regressions on the full modified Rankin Scale (mRS, 0-6) at 90 days, fitted separately for LMRclas (Model 1) and LMRint (Model 2), and two linear regressions on 90-day NIHSS as a continuous measure of neurological severity, fitted separately for LMRclas (Model 3) and LMRint (Model 4). LMRclas and LMRint were entered as their admission values. All four models were fitted on the same 31 patients and adjusted for age, sex, and baseline NIHSS; additional covariates (vascular risk factors, stroke subtype) were not included given the risk of unstable estimates with the available number of events (Peduzzi et al., 1996). Ordinal models are reported as adjusted odds ratios (OR) with 95% confidence intervals; linear models as adjusted β coefficients with 95% confidence intervals. Confidence intervals and p-values were both derived by the Wald method. Model 4 explained 39.0% of the variance in 90-day NIHSS (R² = 0.390; adjusted R² = 0.296; F(4,26) = 4.16, p = 0.010). Sex was coded as male versus female (reference category). The row for the biomarker of interest in each model is shown in bold.*
